# Nondominant Hemisphere Encephalitis in Patient with Signs of Viral Meningitis, New York, USA

**DOI:** 10.3201/eid1508.090466

**Published:** 2009-08

**Authors:** Deborah Asnis, Nadia Niazi

**Affiliations:** Flushing Hospital Medical Center, Flushing, New York, USA

**Keywords:** Herpesviridae infections, herpes simplex, encephalitis, meningitis, brain inflammation, cerebral hemispheres, viruses, New York, USA, letter

**To the Editor:** Herpes simplex virus (HSV) is the most common cause of sporadic fatal encephalitis across the globe and for all ages. HSV is the etiologic agent of 10%–20% of the 20,000 cases of encephalitis per year in the United States (*1*); >50% of untreated cases are fatal. Of the 2 types of HSV, HSV-1 and HSV-2, HSV-1 most commonly affects persons 20–40 years of age, whereas HSV-2 commonly affects neonates. This rapidly progressive disease is a common cause of fatal encephalitis in the United States. Signs and symptoms include fever and headache for a few days, followed by confusion, focal deficits, seizures or hemiparesis, hallucinations, and altered levels of consciousness (*2*). One third of all HSV encephalitis cases afflict children and adolescents. Lumbar puncture typically shows lymphocytic pleocytosis, increased erythrocytes, and elevated protein (*2*); glucose level is typically within normal limits. Serologic assays often show prior infection. Brain imaging frequently indicates unilateral frontal or temporal lobe abnormalities with edema or hematoma (*3,**4*). The involvement of the nondominant brain hemisphere is associated with atypical signs and symptoms (*5*). Diagnosis is usually made by using PCR to examine viral DNA in cerebrospinal fluid (CSF) (*6*). This method of finding DNA in CSF is highly sensitive (98%) and specific (94%–100%). Without therapy, 70% of patients die; with therapy, 20%–30% die (*6*). Illness includes behavioral sequelae.

A 43-year-old female immigrant from China was admitted to Flushing Hospital Medical Center in Flushing, New York, with complaints of headache, fever, and vomiting, which she had experienced for ≈1 week. She had no photophobia, confusion, or rash; neurologic examination found no abnormalities. CSF contained 81 leukocytes with 82% lymphocytes, 3 erythrocytes, protein at 194 mg/dL, and glucose at 67 mg/dL. CSF was positive for HSV-1 viral DNA by PCR. A computed tomography (CT) scan of the head showed unilateral temporal lobe edema. Intravenous acyclovir 10 mg/kg every 8 hours was administered. HIV test was negative. On day 5, a repeat CT scan showed worsening edema and hemorrhage, despite clinical improvement (Figure). CSF contained 490 leukocytes with 99% lymphocytes and protein at 336 mg/dL. After continued treatment with parenteral acyclovir, the patient’s symptoms resolved. On day 12, the patient was discharged after a final CT scan showed resolution of hemorrhage and edema and CSF contained decreased leukocytes and protein.

**Figure:**
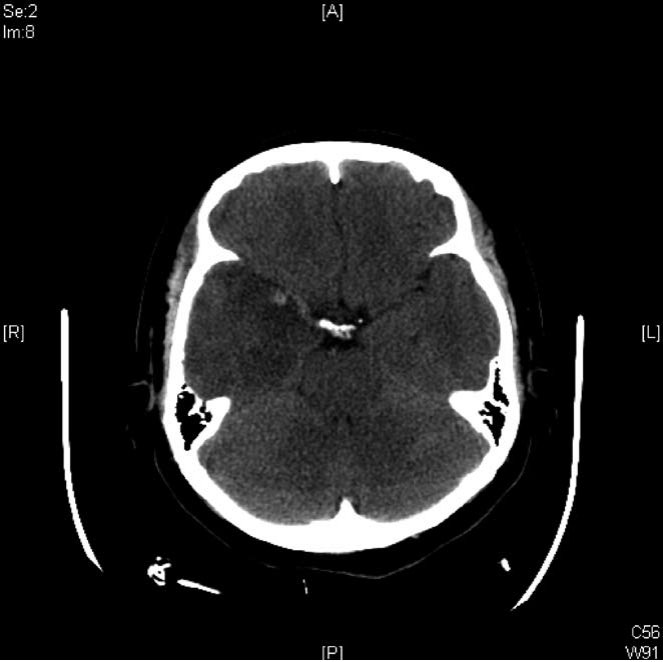
Computed tomography scan showing hemorrhage in edematous part of brain of patient with herpes simplex virus encephalitis, day 5 of hospitalization.

Although this patient had classic signs of meningitis without encephalitis, the CT scan of the head showed cerebral involvement. These factors can be explained by the location of cerebral inflammation in the nondominant lobe of the brain, thereby masking signs of encephalitis. The classic teaching that viral meningitis may not need treatment may miss the occasional viral encephalitis if brain imaging and CSF PCR are not performed. Failure to perform these tests may lead to illness and death from HSV encephalitis if this disease is not considered as a possible diagnosis.
